# Analyzing Patient Secure Messages Using a Fast Health Care Interoperability Resources (FIHR)–Based Data Model: Development and Topic Modeling Study

**DOI:** 10.2196/26770

**Published:** 2021-07-30

**Authors:** Amrita De, Ming Huang, Tinghao Feng, Xiaomeng Yue, Lixia Yao

**Affiliations:** 1 Department of Artificial Intelligence and Informatics Mayo Clinic Rochester, MN United States; 2 Department of Computer Science University of North Carolina at Charlotte Charlotte, NC United States; 3 Division of Pharmacy Practice and Administrative Sciences James L. Winkle College of Pharmacy University of Cincinnati Cincinnati, OH United States

**Keywords:** patient secure messages, patient portal, data model, FHIR, annotated corpus, topic modeling

## Abstract

**Background:**

Patient portals tethered to electronic health records systems have become attractive web platforms since the enacting of the Medicare Access and Children’s Health Insurance Program Reauthorization Act and the introduction of the *Meaningful Use* program in the United States. Patients can conveniently access their health records and seek consultation from providers through secure web portals. With increasing adoption and patient engagement, the volume of patient secure messages has risen substantially, which opens up new research and development opportunities for patient-centered care.

**Objective:**

This study aims to develop a data model for patient secure messages based on the Fast Healthcare Interoperability Resources (FHIR) standard to identify and extract significant information.

**Methods:**

We initiated the first draft of the data model by analyzing FHIR and manually reviewing 100 sentences randomly sampled from more than 2 million patient-generated secure messages obtained from the online patient portal at the Mayo Clinic Rochester between February 18, 2010, and December 31, 2017. We then annotated additional sets of 100 randomly selected sentences using the Multi-purpose Annotation Environment tool and updated the data model and annotation guideline iteratively until the interannotator agreement was satisfactory. We then created a larger corpus by annotating 1200 randomly selected sentences and calculated the frequency of the identified medical concepts in these sentences. Finally, we performed topic modeling analysis to learn the hidden topics of patient secure messages related to 3 highly mentioned microconcepts, namely, fatigue, prednisone, and patient visit, and to evaluate the proposed data model independently.

**Results:**

The proposed data model has a 3-level hierarchical structure of health system concepts, including 3 macroconcepts, 28 mesoconcepts, and 85 microconcepts. Foundation and base macroconcepts comprise 33.99% (841/2474), clinical macroconcepts comprise 64.38% (1593/2474), and financial macroconcepts comprise 1.61% (40/2474) of the annotated corpus. The top 3 mesoconcepts among the 28 mesoconcepts are condition (505/2474, 20.41%), medication (424/2474, 17.13%), and practitioner (243/2474, 9.82%). Topic modeling identified hidden topics of patient secure messages related to fatigue, prednisone, and patient visit. A total of 89.2% (107/120) of the top-ranked topic keywords are actually the health concepts of the data model.

**Conclusions:**

Our data model and annotated corpus enable us to identify and understand important medical concepts in patient secure messages and prepare us for further natural language processing analysis of such free texts. The data model could be potentially used to automatically identify other types of patient narratives, such as those in various social media and patient forums. In the future, we plan to develop a machine learning and natural language processing solution to enable automatic triaging solutions to reduce the workload of clinicians and perform more granular content analysis to understand patients’ needs and improve patient-centered care.

## Introduction

### Background

In the United States, the Medicare Access and CHIP (Children’s Health Insurance Program) Reauthorization Act [1] and *Meaningful Use* program [2] have incentivized the growing adoption of electronic health records (EHRs) and patient health records with the goal of improving the quality of health care delivery systems. Consequently, many health care delivery systems now offer patient portals, tethered to their EHR systems, that allow patients to access their medical records and communicate with their clinicians through secure messages [3]. Patient portals encourage patients to become equal partners in their care and health management and be more engaged and participatory in shared decision making [4]. After the Health Information Technology for Economic and Clinical Health Act was enacted in 2009, patient portals have gained widespread adoption by health care delivery systems in the United States [5,6]. In 2017, more than 90% of the health care delivery systems, including the Veterans Health Administration, Mass General Brigham, Kaiser Permanente, and the Mayo Clinic, offered patient portal access to their patients [7]. Currently, patients send secure web-based messages to request medical appointments and prescription refills [8,9]. Clinicians send patients appointment reminders and promote timely preventive care [10,11]. Patients and clinicians can communicate back and forth easily and in a timely manner about complex situations such as new symptoms, follow-up visits, medication concerns, and medical questions.

With the increase in the number of patients signing up for these portals, the number of secure messages has risen substantially [12-15]. Unfortunately, the content of the large number of patient secure messages in free-text format has not been processed and analyzed systematically and incorporated into the present EHR systems organically to unfold its potential for improving patient-centered care because of technical hurdles. For instance, existing annotated corpora have been mainly developed for sublanguages such as scientific literature in biomedicine and clinical notes; no annotated corpus is available for developing natural language processing (NLP) capabilities in patient-generated formal language. In this study, we propose to develop a data model of health concepts for patient secure messages based on the Fast Healthcare Interoperability Resources (FHIR) standard [16].

A data model is usually made up of entities that represent important items in the domain and relationship assertions among the entities. In our case, a data model will illustrate the key concepts occurring in patient secure messages and the relationships among them. The data model is critical to the development of any information system (eg, a health information exchange system or NLP-based semantic representation system for a patient portal) by providing the definition of the concepts and format of data. Building the factual and useful data model requires a deep understanding of the underlying process and data. Therefore, we also create a large annotated corpus for analyzing the contents of sampled patient secure messages to better understand patients’ concerns. Once complete, we further apply topic modeling techniques independently to investigate whether the patients’ focuses and concerns in 3 common medical conditions align with the developed data model. We build the annotated corpus primarily to build the data model, and topic modeling can serve as an independent and primitive validation of the data model. We choose topic modeling instead of information extraction because we build the annotated corpus primarily to build the data model. Topic modeling, as an unsupervised method, generates results independently of the corpus and thus can serve as an independent validation of the data model. We expect a much more rigorous evaluation and validation by building collaborations and partnerships with domestic and international researchers in the field.

With all the necessary preparation, our ultimate objective is to develop an NLP system that will automatically identify and extract significant information from unstructured patient secure messages for the purpose of automatically triaging patient secure messages, reducing the workload of clinicians by chatbot, and performing more granular and sophisticated content analysis to understand patients’ needs and improve shared decision making and patient-centered care.

### Related Work

As patient secure messages are relatively new, very little research has focused on automatically identifying and standardizing their content despite their important implications. North et al [17] analyzed the content of 6430 secure messages to assess the overall risk associated with the messages and to determine whether patients were using portal messages for symptoms requiring urgent evaluation. Their study showed that patients used portal messages 3.5% of the time for potentially high-risk symptoms of chest pain, breathing concerns, abdominal pain, palpitations, lightheadedness, and vomiting. Sulieman et al [18] also developed machine learning models on patient portal secure messages regarding surgical issues to identify message threads that involve medical decision making from a health care provider and to classify the complexity of the decision. Cronin et al [19] built patient portal message classifiers using rule-based and NLP techniques such as the bag-of-words model. They curated a gold standard data set of 3253 portal messages annotated by communication types such as informational, medical, logistical, and social. This study also focuses on developing a data model—a standard framework—to address the issues of content analysis, information extraction, and integration of significant information from patient secure messages leveraging Health Level-7 (HL7) FHIR.

HL7 is a nonprofit standard development organization accredited by the American National Standards Institute, and it is dedicated to providing a comprehensive framework and related standards for the data exchange, integration, sharing, and retrieval of electronic health information [20]. FHIR is an improved health data exchange standard that comprehensively defines how information can be exchanged among different systems regardless of how it has been stored and allows health care information to be accessible to those who need it for the benefit of health care quickly and easily [21]. Instead of traditional document-centric approaches, HL7 FHIR takes a modular approach and represents atomic or granular health care data (eg, heart rate, procedure, medication, and allergies) as independent modular entities, concepts, and actions involved in health care information analysis, exchange, and integration as resources [22]. Existing studies on developing data standards have mostly focused on analyzing, extracting, and integrating structured data from EHRs, mobile-based patient health records, and medical apps. In 2018, Hong et al [23] for the first time introduced a scalable and standard-based framework for analyzing and integrating both structured and unstructured EHR data by leveraging the FHIR specification [23]. The scope and use of the FHIR framework do not completely meet the requirements of our study. We aim to develop an HL7 FHIR–based data model that precisely analyzes and extracts patient secure messages.

## Methods

### Overview

**Figure 1:**
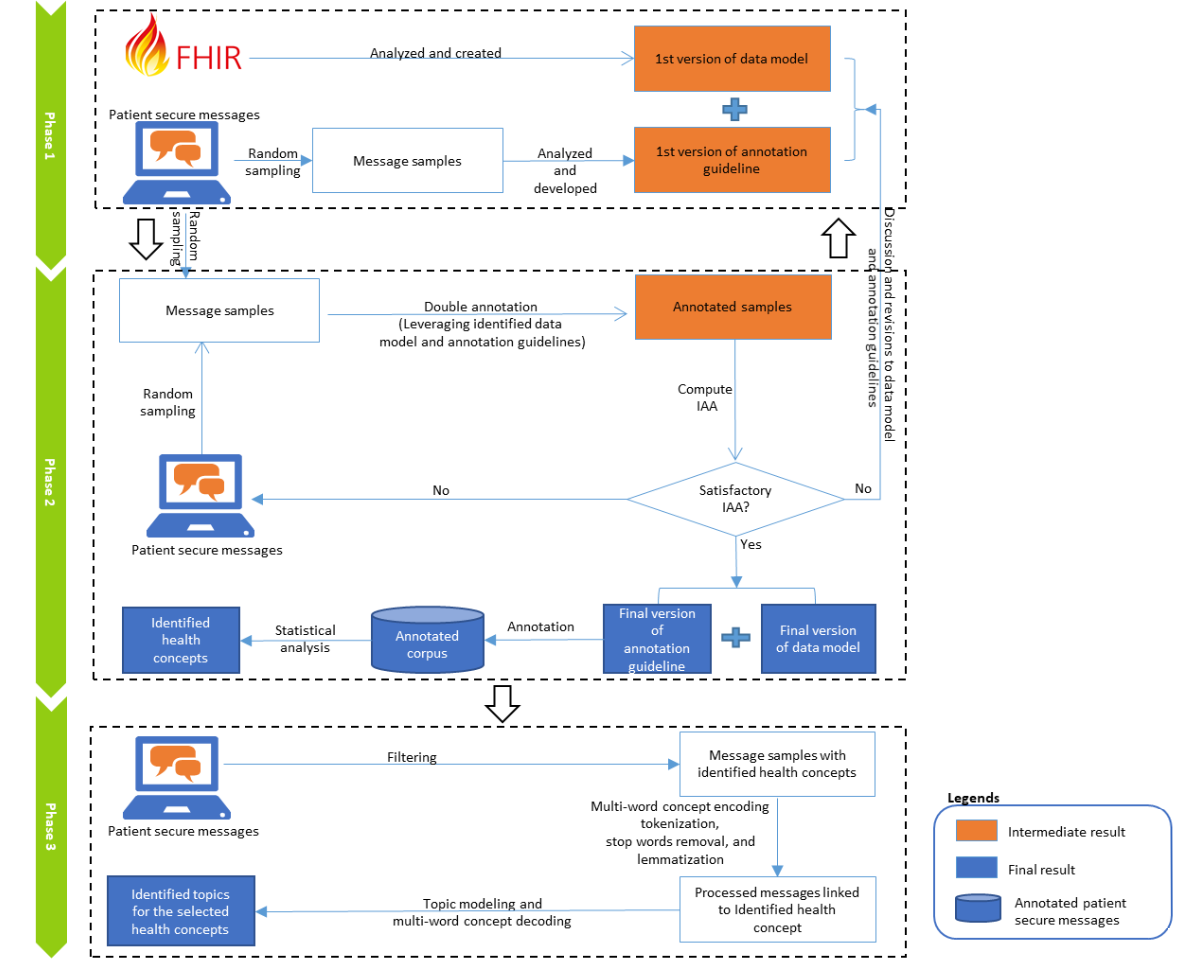
The workflow for developing the data model for patient secure messages, annotated corpus, and topic modeling analysis. FHIR: Fast Healthcare Interoperability Resources; IAA: interannotator agreement.

A 3-phase workflow for developing the data model and annotated corpus is shown in Figure 1. We collected more than 2 million patient-generated secure messages from Mayo Clinic Patient Online Services [24]. We developed the first draft of the data model and annotation guideline by analyzing FHIR and manually reviewing 100 sentences from the sampled secure messages. We then randomly selected, annotated, and examined additional sets of 100 sentences from the secure messages to iteratively update the data model and annotation guideline until interannotator agreement (IAA) was achieved. Subsequently, we created the annotated corpus by annotating 1200 sentences from the randomly selected 2100 sentences. Finally, we calculated the frequency of the identified health concepts in the annotated corpus and performed topic modeling to extract hidden topics of all the patient secure messages linked to frequently mentioned health concepts. In the following sections, we will discuss data collection and preprocessing, design of the data model, development of the annotation guideline, creation and analysis of the annotated corpus, and topic modeling in more detail. The entire data model can be found in Multimedia Appendix 1. The details of data set collection and processing and annotation and topic modeling and annotation guideline can be found in Multimedia Appendix 2 [16,24-33] and Multimedia Appendix 3 [1-3,16,24].

### Ethics Approval

No patient was exposed to any intervention. We used data from the Mayo Clinic Unified Data Platform to develop the annotated corpus and for the analysis. The study was approved by the Mayo Clinic Institutional Review Board (19-002211).

### Data Set Collection and Preprocessing

The Mayo Clinic Rochester started the patient portal (Patient Online Services) in 2010 for primary care practice and later extended it to specialty practice in 2013 [24]. We collected more than 2 million patient-generated secure messages from the online patient portal between February 18, 2010, and December 31, 2017. We removed messages with empty message bodies. Each message has a unique message ID, previous message ID, initial message ID, sender ID, recipient ID, date and time of the message creation, message subject, and message body. As we have mentioned earlier, the details are in Multimedia Appendix 2.

### Design of a Data Model for Patient Secure Messages

Creating a data model for unstructured patient narratives is a very challenging task. After the literature review and initial analysis of the sampled secure messages, we decided to use the FHIR standard to develop the data model (Multimedia Appendix 1) because it comprehensively represents the modular entities, concepts, and actions involved in health care information exchange.

FHIR defines a hierarchical set of core and infrastructure resources for handling health concepts in an EHR [34]. After analyzing version 4 of the FHIR standard [16] and the sampled secure messages, we generated the first draft of the data model with 3 hierarchical levels—macroconcepts, mesoconcepts, and microconcepts—to extract information from the patient secure messages. We merged and revised some concepts from FHIR after analyzing the messages. For example, we merged 2 similar but separately defined top-level concepts (foundation concept and base concept) in FHIR into 1 macroconcept (foundation and base concept) in the data model. We also introduced a microconcept—*unspecified—*as an attribute under all mesoconcepts. *Unspecified* refers to the general terms under most of the mesoconcepts that cannot be categorized into any specific microconcepts. We deleted some mesoconcepts under clinical macroconcepts such as *clinical impression*, *detected issue*, *medication knowledge*, *molecular sequence*, and *care team* because they are not relevant to patient secure messages. The data model also underwent rounds of revisions during the annotation process to handle inconsistencies, and disagreement occurred between the annotators. For instance, in the first draft of the data model, the mesoconcept *patient* has 7 microconcepts following FHIR, such as *identifier*, *name*, *telecom*, *gender*, *birthdate*, *address*, and *marital status*. In the final data model, the mesoconcept *patient* has 4 microconcepts, such as *unspecified*, *privacy*, *lifestyle,* and *diet,* to better understand patients’ medical records and history. All personal information related to the patient is kept under *privacy* to maintain data privacy.

### Development of an Annotation Guideline

For any annotation task, it is important that all annotators follow the same standard (annotation guideline) to minimize annotation confusion and errors. We manually reviewed several sets of 100 randomly selected message sentences to develop an annotation guideline. More specifically, we created the first version of our annotation guideline by analyzing the first set of 100 message sentences. Subsequently, our annotators independently annotated another set of 100 message sentences to examine the effectiveness of the annotation guideline until the annotators reached a considerable amount of agreement.

The final annotation guideline consists of 2 sections: (1) the first section discusses the general annotation rules; (2) the second section describes the health concepts and associative rules for the identification and extraction of these concepts together with specific examples. For instance, it is challenging to differentiate 2 microconcepts (*name* and *symptoms*) under the mesoconcept *condition*. The *condition-name* microconcept refers to the name of a disease and/or medical condition (eg, *rheumatoid arthritis*, *diabetes mellitus*, and *influenza*). The *condition-symptom* microconcept denotes a physical or mental feature or symptom (eg, *sore bottom* and *numb arm*) of a disease and/or medical condition. As per general annotation rules, all modifiers (eg, adjectives and adverbs) and possessives are removed for annotation to make text spans consistent. For the cases *special calcium pill*, *this medicine,* and *my rheumatoid arthritis*, the modifiers and possessives *special*, *this,* and *my* are not considered for annotation. All patient private information (eg, name, identity number, and clinic number) is not disclosed to maintain data privacy. The guideline clearly defines the concepts and rules to lessen the scope of ambiguity and error in the annotation and increase the possibility of agreement among the annotators to develop a quality corpus. The complete guideline for annotating patient secure messages is listed in Multimedia Appendix 3.

### Development and Analysis of an Annotated Corpus of Patient Secure Messages

We chose the Multi-purpose Annotation Environment tool to annotate patient secure messages because of its ease of use and ability to fix discrepancies [25]. The Multi-purpose Annotation Environment tool requires a document type definition file with concept tags and attributes. We chose 2 professional annotators—a clinically trained linguist and a student pharmacist—to create a standard error-free corpus. They initially analyzed and revised several sets of 100 randomly sampled message sentences to iteratively improve the data model and annotation guideline. To check the consistency between the annotators, we calculated the F1 score of 2 separate annotation sets as the IAA score using General Architecture for Text Engineering software [26]. The IAA scores were computed using the F1 score as a criterion at the level of entities. This helped us to understand the span of the concepts on which the annotators agreed, disagreed, and partially agreed. We decided to follow the lenient parameter for measuring the IAA. With the lenient approach, the annotations that overlap are counted as a partial match, in contrast with the strict approach in which the annotations have to match with one another completely. We can consider this sentence as an example: “My mother has severe sinus headaches for several months.” Now, if one annotator annotates “sinus headache” and another annotates “severe sinus headache,” then the strict IAA approach will give us no match, but the lenient approach will consider this as an overlap. The F1 scores of the first set of annotations were 0.42 (macro mean) and 0.67 (micro mean). After discussing and resolving the disagreements, the annotators annotated another set of the same sentences, and the F1 scores were quite satisfactory: 0.62 (macro mean) and 0.74 (micro mean). Our annotators finally developed a quality corpus of 1200 randomly selected sentences.

After annotation, we performed summary statistics to calculate the frequencies of the identified health concepts (ie, macroconcepts, mesoconcepts, and microconcepts) in the annotated corpus of patient secure messages. The distribution of these health concepts helps us to understand which concepts patients were mostly concerned about and communicated to their health care providers. The details are provided in Multimedia Appendix 2.

### Topic Modeling

After analyzing the annotated corpus, we selected 3 health microconcepts (ie, fatigue, prednisone, and patient visit) as representative cases for topic modeling analysis. The chosen microconcepts and the corresponding meso concepts and macroconcepts were frequently discussed in the patient portal messages (refer to the *Results* section for more details). Fatigue is an instance of a top-mentioned microconcept, symptom, under the condition mesoconcept and clinical macroconcept. Prednisone is a case of a microconcept, name, under the medication mesoconcept and clinical macroconcept, about which patients have expressed most concern in the patient secure messages. Patient visit is an example of a largely discussed microconcept, type, under the appointment mesoconcept and the foundation and base macroconcept.

After multi-word concept encoding and health concept recognition using MetaMap [27], we collected 41,490, 27,743, and 95,533 patient secure messages that mentioned the health microconcepts fatigue, prednisone, and patient visit, respectively, to examine the focus of those messages. MetaMap is a highly configurable program. It has been developed by Dr Alan Aronson at the National Library of Medicine to map biomedical texts to the Unified Medical Language System.

Topic modeling automatically identifies topics or themes in a large collection of documents in terms of a set of keywords that occur together and most frequently [35,36]. We used latent Dirichlet allocation (LDA) [28], a state-of-the-art unsupervised topic modeling method as implemented in Machine Learning for Language Toolkit [29], to learn the hidden topics of patient secure messages related to each of the 3 health microconcepts. After tokenization, stop word removal, and lemmatization, each patient secure message was converted into a vocabulary vector where the elements were the frequency of each lemma (including Concept Unique Identifier encoded by MetaMap) without considering the order of the lemma.

We quantitatively calculated topic coherence [30] and asked domain experts to qualitatively evaluate the learned topics. More specifically, we evaluated the topic coherence at different topic numbers (ie, 2, 4, 6, 8, 10, 12, 14, 16, 18, 20, 22, 24, 26, 28, and 30) to determine the optimal topic number for the 3 selected health microconcepts. We found that the optimal topic number was 8 for fatigue-related messages, 10 for prednisone-related messages, and 12 for patient visit–related messages. We also investigated the hyperparameters of and in the LDA. controls the topic distributions over a document. A smaller results in fewer topics that are statistically associated with a document. determines the word distributions over a topic. A smaller leads to fewer words that are statistically linked to a topic. We set the automatic optimization of the hyperparameters of and in Machine Learning for Language Toolkit (Mallet) during topic modeling.

## Results

### Data Model for Patient Secure Messages

The data model for patient secure messages has a 3-level hierarchical structure consisting of 3 macroconcepts, 28 mesoconcepts, and 85 microconcepts. Textbox 1 provides a partial illustration of the data model (refer to Multimedia Appendix 1 for the full data model). The 3 macroconcepts in the data model are the foundation and base macroconcept, clinical macroconcept, and financial macroconcept. Foundation and base concepts are the basic infrastructure of the health care system concepts on which the rest of the specifications are built. The mesoconcepts of the foundation and base macroconcept include patient, practitioner, related person, organization, health care service, device, appointment, encounter, and document reference. Clinical macroconcepts refer to core clinical components, including allergy intolerance, adverse event, body structure, specimen, condition, procedure, family member history, observation, laboratory test, imaging, medication, immunization, care plan, care team, referral, and risk. The financial macroconcepts cover all finance-related issues such as coverage eligibility, claim payment, account, and explanation of benefits. All the mesoconcepts have been further categorized into microconcepts as attributes, and each mesoconcept has an *unspecified* microconcept. For example, as shown in Textbox 1, the mesoconcept *patient* is categorized as *unspecified*, *privacy*, *lifestyle*, and *diet*. The mesoconcept *coverage-eligibility* has 5 microconcepts, including *unspecified*, *percentage*, *insurance ID*, *benefit category*, *insurer*, and *insurance*.

**A partial illustration of the data model for patient secure messages.**

**Health Care System Concepts and Their Definitions**

Foundation and base concepts

- Patient—Demographic and other administrative information about an individual receiving health care services
  Unspecified
  Privacy
  Lifestyle
  Diet
- Appointment—A booking of a health care event between patients and practitioners (or other related persons or devices) on a specific date at a specific time
  Unspecified
  Status
  Type
  Reason

Clinical concepts

- Specimen—A sample taken from a biological entity for laboratory analysis
  Unspecified
  Name
- Laboratory test—Tests (eg, clinical, hematological, or microbiology tests) performed on patients and groups of patients and the results derived from the tests
  Unspecified
  Name
  Result

Financial concepts

- Coverage eligibility—Information on patients, insurers, insurance, coverage, plan details, reimbursement, and payment for health care services
  Unspecified
  Percentage
  Insurance ID
  Benefit category
  Insurer
  Insurance

- Explanation of benefits—Information on claim details and adjudication details from the processing of claims
  Unspecified

### Annotated Corpus of Patient Secure Messages

**Figure 2:**
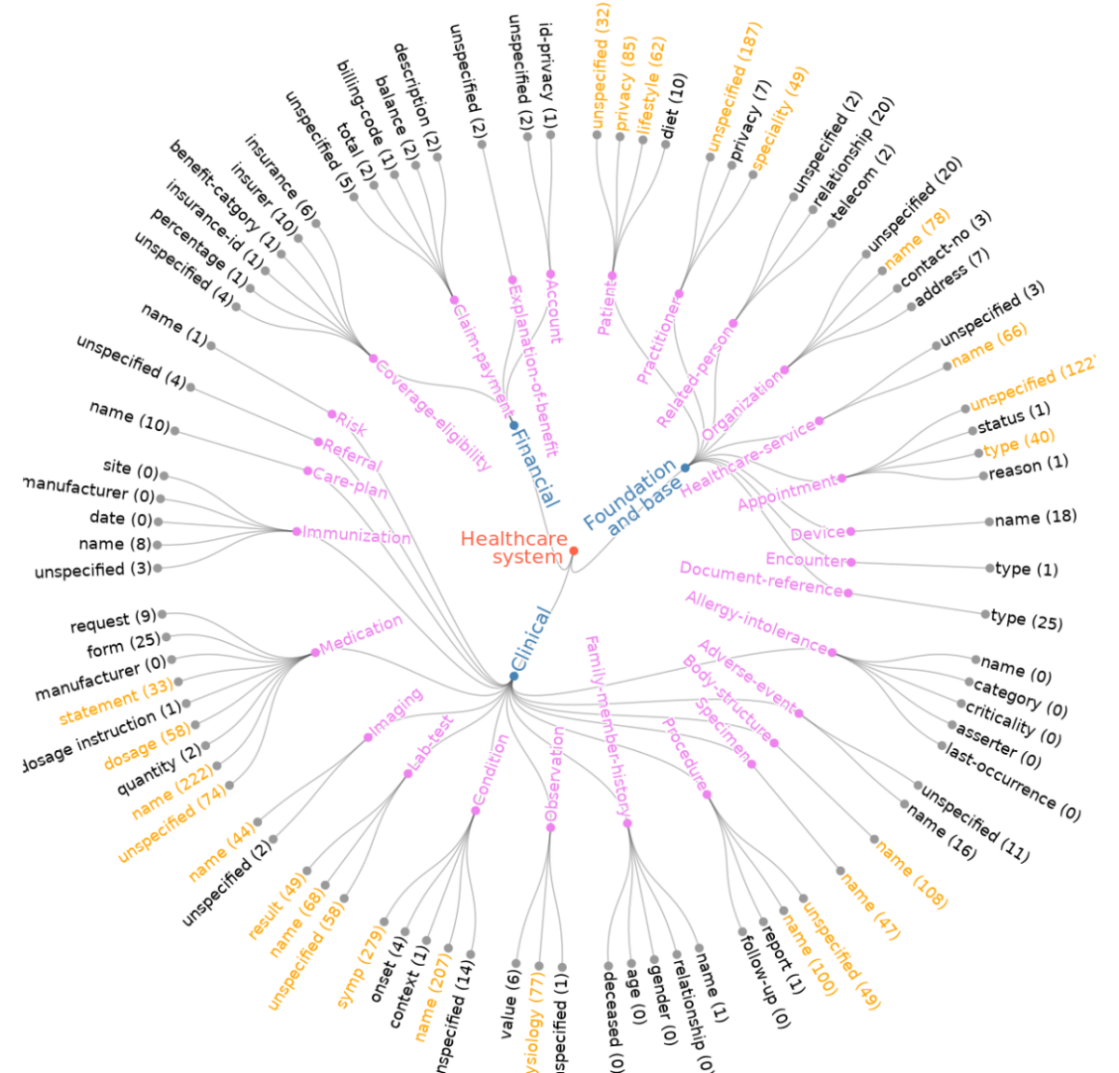
Radial tree to illustrate hierarchical health concepts in the annotated corpus.

We annotated 1200 sentences of patient secure messages based on the data model. Figure 2 illustrates the frequency of hierarchical health concepts in the annotated corpus. The concepts in blue, purple, and black represent macroconcepts, mesoconcepts, and microconcepts, respectively. The concepts in orange illustrate the most frequent microconcepts along with their occurrences in the annotated text. Foundation and base macroconcepts make up 33.99% (841/2474) of the annotated corpus, clinical macroconcepts make up 64.38% (1593/2474) of the annotated corpus, and financial macroconcepts make up 1.61% (40/2474) of the annotated corpus, respectively. Patients shared some information about insurance, coverage, and payments in the secure messages. Among the 28 mesoconcepts, the most discussed were condition (505/2474, 20.41%), medication (424/2474, 17.13%), practitioner (243/2474, 9.82%), patient (189/2474, 7.63%), laboratory test (175/2474, 7.07%), appointment (164/2474, 6.62%), procedure (150/2474, 6.06%), and organization (108/2474, 4.36%). The most frequently used microconcepts were various condition names (eg, fatigue), medication names (eg, prednisone), and appointment types (eg, patient visit).

### Topics of Patient Secure Messages

**Figure 3:**
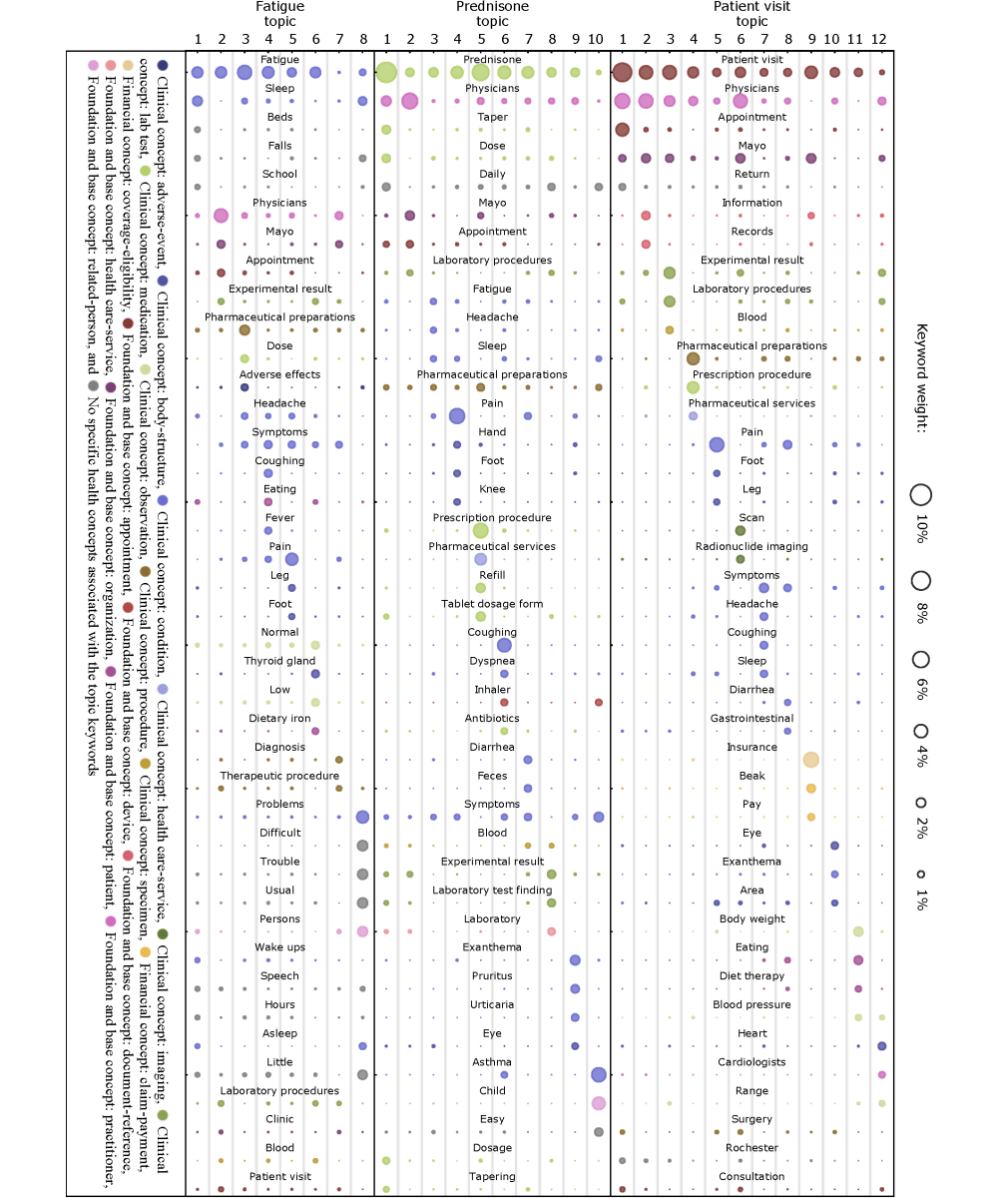
The distribution of 40 keywords representing different topic themes (topic 1-12) for the microconcepts fatigue, prednisone, and patient visit. The color indicates different health concepts linked to the topic keywords.

After identifying the frequently discussed health concepts, we used a topic modeling technique to discover the latent topics in terms of a group of keywords for patient secure messages mentioning fatigue, prednisone, and patient visit. As shown in Figure 3, we highlight 8, 10, and 12 meaningful topics of patient secure messages related to fatigue, prednisone, and patient visit, respectively. We use a color scheme to represent the top health microconcepts in the topics of patient secure messages associated with fatigue, prednisone, and patient visit. More topic keywords and details can be found in Multimedia Appendix 4. Among the 120 top keywords, 107 (89.2%) were found to be health concepts in the data model. The concepts that were not mapped include general English words such as *hours*, *trouble*, and *difficult*.

#### Fatigue

Fatigue is a common clinical condition discussed by patients in the patient secure messages. Topic modeling on patient secure messages targeting fatigue showed that most of the patient discussions were centered around sleepiness, adverse drug effects, and relevant symptoms and conditions. More specifically, the keywords *sleep*, *beds*, *fall*, *asleep*, *little*, and *wake ups* frequently appeared in these patient secure messages, which highlighted the strong association between fatigue and sleepiness. The topic words *pharmaceutical preparations*, *dose*, *adverse effects*, and *headache* revealed that fatigue might be an adverse effect related to some drugs. Other relevant symptoms and conditions such as *headache*, *coughing*, *fever*, and *pain* were also found in patient secure messages discussing fatigue. After tracing back to the original patient secure messages, we also found mentions of *low*, *thyroid gland*, and *dietary iron*. This finding is consistent with studies reporting that fatigue is one of the most common signs of an underactive thyroid (hypothyroidism) [37] and iron deficiency [38].

#### Prednisone

Prednisone is a corticosteroid drug that controls inflammation by suppressing the human immune system. Prednisone appeared frequently in the patient secure messages. The focus of patient secure messages related to prednisone was primarily on drug use, side effects, and disease treatment. The topic words related to prednisone use and dose such as *taper*, *dose*, *daily*, *dosage*, and *tapering* were mentioned most often. Prednisone has been used to treat a variety of medical conditions, as shown by the keywords such as *cough*, *dyspnea*, *asthma*, *pruritus*, and *urticaria* [39-42]. In addition, the patients reported side effects such as *fatigue*, *headache*, *sleep* and *pain* after taking prednisone [43].

#### Patient Visit

Patient visit is a type (attribute) of patient *appointment* under the *foundation and base* concept. The topics of patient secure messages related to patient visits revealed various potential reasons for patient visits by making appointments. For example, many patients requested appointments to visit the Mayo Clinic by mentioning their health information and medical records (*mayo*, *information*, and *records*), laboratory tests and test results (*experimental result*, *laboratory procedure*, *blood*, *scan*, and *radionuclide imaging*), and medications (*pharmaceutical preparation*, *prescription procedure*, and *pharmaceutical services*). The keywords *pain*, *symptoms*, *headache*, *coughing*, *diarrhea*, *exanthema*, *heart*, and *cardiologists* suggest the purpose of their visit to the Mayo Clinic. In addition, some patients made appointments for financial issues indicated by the topic words *insurance* and *pay*.

## Discussion

### Principal Findings

Patient secure messages contain valuable information about the quality of health care delivery systems, drug efficacy and safety signals, and other pain points of patient health management. The data model and annotated corpus enable us to extract information from patient secure messages for systematic content analysis and to resolve the challenges of data analysis and integration by standardizing the unstructured data with the structured data system. In future, this data model can be used and extended to model patient narratives from social media platforms [44] to analyze their content. Our future aim is to leverage this data model and annotated corpus for developing machine learning models and NLP-enabled parsing tools for automatically parsing patient narratives to advance clinical research and practice [45,46].

During annotation, we faced some challenges because of the heterogeneous and superficial nature of the language used in the messages, which often deviated from formal English grammar, spelling, and punctuation rules. These challenges can often lower the quality of the data and make them less accessible to automated processing by a system. We discussed and designed some solutions to overcome these challenges. The challenges and their solutions are discussed in detail in Multimedia Appendix 2. During the comparison between the 2 sets of the first set of annotation, the level of agreement between the annotators was consistent for a few subconcepts, such as *appointment*, *diagnostic report*, *immunization*, *medication*, and *practitioner.* In contrast, it was very unsatisfactory for subconcepts such as *specimen*, *related-person*, *document-reference*, *eligibility*, and *health care-service*. Although the 2 annotators achieved a satisfactory IAA score later, annotation bias likely exists in the annotated corpus.

After analyzing the annotated corpus and identifying important health concepts in patient secure messages, we further used topic modeling to automatically uncover hidden topics of patient secure messages mentioning health concepts [47]. In this way, we were able to evaluate the data model and understand the focus of patient discussion and concerns in the patient secure messages. For example, prednisone, because it is a corticosteroid drug that controls inflammation, was a highly discussed topic in the patient secure messages. The patient-provider discussion on prednisone was primarily centered around medication use, side effects, and disease treatment [39-43]. These findings offer useful information for shared clinical decision making and patient-centered care. LDA exploits statistical inference to identify latent topics using a bag-of-words model and term frequency. Therefore, LDA mainly discovers topics with high frequency and dominant terms and pays little attention to rare, yet meaningful, topics from patient secure messages. In addition, hypermeter tuning in LDA can be more art than science.

Our study is the first to develop a data model based on HL7 FHIR to understand and analyze the content of patient secure messages. This study has several limitations. For annotation, the F1 scores were 0.62 (macro mean) and 0.74 (micro mean). This indicates that the task of annotation is a difficult one because we need to assign 3-level hierarchical health concepts to an identified health entity, and there are 85 microconcepts to be selected in the data model. Our annotators not only need to assign a category to these potential entities but also to identify their boundaries. If the boundaries are defined differently by different annotators, it is considered *not* *consistent*. The language used in patient secure messages to describe medical concepts is casual, colloquial, and ambiguous. We revised our annotation guideline 5 times and trained our annotators 4 times. We believe that the F1 score could be improved, given more resources for annotation.

All the messages analyzed in this study were sent by patients. We did not analyze the messages generated by the clinicians who read and replied to these patient secure messages. The Mayo Clinic at Rochester, Minnesota, is a large nonprofit academic medical center that provides comprehensive patient care in the United States. We acknowledge that the patients and medical practices at the Mayo Clinic are not necessarily a representative cross-section of all patients and medical practices in the country. It is also unknown how the data model can be applied to other hospitals in other countries, given different models of care and patient-clinician relationships. For example, we are aware that in China, there are no patient portals, and patients communicate with their providers through other means. Thus, we share our model, annotation guideline, and other materials with the broader scientific community and welcome all sorts of collaboration or partnership.

Finally, we acknowledge that evaluation and validation are key and challenging in this case. Strictly speaking, the data model can only be validated for the claimed utilities in real-world implementations. The topic modeling analysis we conducted was only an initial and weak evaluation. We expect to conduct a much more rigorous evaluation and validation by building collaborations and partnerships with domestic and international researchers in the field and by moving forward with building the NLP systems.

In the next stage of our study, we aim to curate a larger annotated corpus and use it for training and testing machine learning models for automated triaging. The manually annotated corpus is reusable for future NLP research to save manual effort and cost, although there might be some challenges related to its reuse because of data privacy and confidentiality challenges. This corpus is also generated based on patient secure messages; therefore, there always will remain a question of its usability in different domains of medical research.

### Conclusions

A patient portal as a tethered EHR system enables patients to access their medical records, seek support, and share their opinions with their caregivers through secure messaging between their clinical visits. The large volume of secure messages opens new opportunities and challenges for understanding patient concerns and information integration into EHRs to improve patient-centered care. This study is a novel attempt to identify the content of patient secure messages based on the foundation and base, clinical, and financial concepts of HL7 FHIR standards. The data model and annotated corpus enable us to meet the challenges of analyzing and understanding unstructured health information from patient secure messages along with the topic modeling technique to discover the hidden topics on interesting health concepts in patient secure messages.

The data model could be potentially used for automatically identifying and analyzing other types of patient narratives such as those in various social media and patient forums. In the future, we plan to develop a machine learning and NLP solution to enable automatic triaging solutions to reduce the workload of clinicians and perform more granular content analysis to understand patients’ needs and improve patient-centered care.

## Appendix

Multimedia Appendix 1 Data model for patient secure messages based on Health Level-7 Fast Healthcare Interoperability Resources.

Multimedia Appendix 2 Description of methodology in detail.

Multimedia Appendix 3 Annotation guideline.

Multimedia Appendix 4 Results of topic modeling.

## Abbreviations

EHR: electronic health record
FHIR: Fast Healthcare Interoperability Resources
HL7: Health Level-7
IAA: interannotator agreement
LDA: latent Dirichlet allocation
NLP: natural language processing
