# Supplementary material for: Analyzing Patient Secure Messages Using a Fast Health Care Interoperability Resources (FIHR)–Based Data Model: Development and Topic Modeling Study
Source: J Med Internet Res. 2021 Jul 30;23(7):e26770. doi: 10.2196/26770 (PMC8367168; doi:10.2196/26770)
Supplement: Multimedia Appendix 2 [file jmir_v23i7e26770_app2.pdf]

# Analyzing Patient Secure Messages Using a Fast Health Care Interoperability Resources–Based Data Model: Development and Topic Modeling Study

## Multimedia Appendix 2

Amrita De[†1], Ming Huang[†1], Tinghao Feng[2], Xiaomeng Yue[3], and Lixia Yao[*1]

[1]Department of Artificial Intelligence and Informatics, Mayo Clinic, Rochester, MN, United States

[2]Department of Computer Science, University of North Carolina at Charlotte, Charlotte, NC, United States

[3]Division of Pharmacy Practice and Administrative Sciences, James L. Winkle College of Pharmacy, University of Cincinnati, Cincinnati, OH, United states

[†] Equal contribution

**Corresponding Author:**

**Lixia Yao, PhD**

All authors have no conflict of interest to declare.

With the exponential growth in health data, it becomes important to identify and analyze the content of patient secure messages in online patient portal along with the EHR data. Prior to our study, very few researchers have studied the content of patient secure messages for enabling automatic and semi-automatic triaging solutions. If we can classify and extract significant information from this huge amount of data, we will be able to minimize the burden of clerical work at the back end and provide clinicians a comprehensive summary of the patient's need to improve the quality of patient-centered healthcare. In our study, we create a data model of healthcare concepts based on Health Level Seven (HL7) standard Fast Healthcare Interoperability Resources (FHIR) [1] and a large annotated corpus in order to better understand the content of patient secure messages and their concerns. We further apply topic modeling techniques to investigate if the patients' focuses and concerns in 3 common medical conditions align with the developed data model. We have built the annotated corpus primarily to develop the data model and the topic modeling can serve as an independent and imperfect validation of the data model. In future we aim to develop machine learning models and NLP tools for automatically parsing patient secure messages to improve the quality, safety and efficiency of patient care and reduce health disparities We believe our data model for patient secure messages can be used also as a standard framework for automatically identifying and extracting information from other types of unstructured patient narratives in social media and patient forums. The detailed steps for developing data model and annotated corpus and topic modeling are discussed below.

**Technical steps to develop Data Model for Patient Portal Secure Messages and Annotated Corpus and Topic Modeling for training and testing Machine learning Model**

We collected over 2 million patient secure messages from Mayo Clinic Patient Portal Services [2] to develop and evaluate the data model and an annotated corpus. Our present study focuses on developing a data model, a standard framework to identify and understand the content of patient secure messages and generate an annotated corpus and topic modeling to further validate the data model. We will discuss the methodology in details including dataset collection and preprocessing, creation of data model, development of annotation guideline, annotation process and calculation of inter-annotator agreement, analysis of annotated corpus and topic modeling to learn hidden topics of patients' concern.

## 1. Dataset Collection and Preprocessing

Patients often use secure messaging in patient portals to timely communicate with their providers for a wide range of health issues. Patients and clinicians can communicate back and forth on complex situations such as new symptoms, disease follow-ups, medication concerns and medical questions termed as e-visits [3]. The analysis and adoption of patient portal content have become increasingly important due to its potentials to promote patient engagement and improve self-management and decision-making for further healthcare. But unfortunately, this large amount of patient secure messages from online portal have not been analyzed systematically due to the lack of data standard, privacy, lack of health literacy, lack of integration and interoperability. Our study aims to analyze and extract this patient portal secure messages.

Online Patient Portal service in Mayo Clinic [2], Rochester started in 2010 for primary care practice and then it was extended to specialty practice in 2013. We collected more than 2 million patient secure messages from the portal between February 18, 2010 and December 31, 2017. The secure messages are sent from patients to providers. Each message has unique message ID, initial message ID, sender ID, recipient ID, date and time of the message initiated, message subject and message text body. Patients' privacy and confidentiality have been prioritized. In patient secure messages, patients, other family members and caregivers share and inquire about health condition, the appointments, diagnosis, treatment procedure, test results, medication history, medical bill and insurance policy. They also share sometimes their personal experiences about the care they receive from the healthcare providers.

We used Python Pandas library for preparing this data for further analysis. All the individual posts were broken into sentences and we randomly selected 2100 sentences for our initial study. We removed all the sentences without any medical or clinical information or irrelevant to our study. The descriptive statistics of the corpus such as the average length of each message and the average number of entities annotated in each message are shown below in table 1.

Table 1: Statistics of annotated corpus (1200 patient secure message sentences)

| | Minimum | Maximum | Mean | Median |
|---|---|---|---|---|
| Number of characters in a message sentence | 10 | 522 | 90.6 | 80 |
| Number of words in a message sentence | 2 | 94 | 16.7 | 15 |
| Number of health concepts annotated in a message sentence | 0 | 15 | 2.1 | 2 |

The 1200 annotated message sentences were randomly selected from 399 patient secure messages, which were sent by 399 unique patients. The following table 2 and 3 shows the age and gender breakdown of those patients. Given that the age and gender information was voluntarily reported by patients themselves and in this study we are not able to link the patient portal data to the EMR database due to the default setting and study protocol constraint, we are not able to further analyze more demographic and diagnosis information of those patients.

Table 2: Statistics of Pateints' Age

| Total Patients 399 | | |
|---|---|---|
| Age | Number | Percentage |
| 18-29 | 23 | 5.764411 |
| 30-39 | 42 | 10.526316 |
| 40-49 | 46 | 11.528822 |
| 50-64 | 75 | 18.796992 |
| 65+ | 64 | 16.040100 |
| <18 | 32 | 8.020050 |
| Unknown | 117 | 29.323308 |

Table 1: Statistics of Pateints' Gender

| Total Patients 399 | | |
|---|---|---|
| Gender | Number | Percentage |
| Female | 180 | 45.112782 |
| Male | 103 | 25.814536 |
| Unknown | 116 | 29.072682 |

## 2. Design the Data Model for Patient Secure Messages

A data model of hierarchical concepts enables us to identify and extract important health information from patient secure messages in a free text format. Creating a data model for unstructured patient narratives is a very challenging task. After the literature review and the initial analysis of the sampled secure messages, we decided to use HL7 FHIR standard to develop the data model for patient secure messages (DM4PSM). The FHIR data standard represents all the healthcare related concepts in a hierarchical structure. HL7 FHIR data standard has been classified under five broad categories as foundation, base, clinical, financial and specialized [1]. We adopted and revised the recent released version of FHIR [1] in order to meet the scope and challenges of our study. We adopted founadation, base, clinical and financial concepts and merged foundation and base concepts as a single concept. At the initial level of our

study, we analyzed the formats and elements published by FHIR to generate the first draft of data model and gradually it went through some iterative process of revisions. Finally, we have a data model of three hierarchical levels with 3 macro concepts (foundation and base concept, clinical concept and financial concept), 28 meso concepts and 85 micro concepts. Foundation and base concepts are the basic infrastructure of a healthcare system on which rest of the specification is built. They include patient, practitioner, related-person, organization, healthcare-service, appointment, device, encounter and document-reference. Clinical concepts are those concepts referring to core clinical concepts such as allergy intolerance, adverse event, body-structure, specimen, condition, procedure, family-member history, observation, lab-test, imaging, medication, immunization, care-plan, care-team, referral and risk. The financial concepts cover all the financial transactions which happen between a healthcare provider, patient and insurer such as coverage-eligibility, claim-payment, account and explanation of benefits. All the concepts have been defined based on FHIR data standards and their definitions. We didn't adopt all the foundation and base concepts as meso and micro concepts and added few micro concepts in the data model for better understanding the patient secure messages. For example, the meso concepts 'patient' under foundation and base macro concept has been further classied as 'privacy', 'lifestyle' and 'diet'. We have deleted and merged some meso concepts in our data model as well. HL7 FHIR has six healthcare concepts related to medication under clinical conepts (i.e., medication request, medication administration, medication dispense, medication statement, medication, and medication knowledge). We merged all these six concepts under medication meso-concept and further classified it with some attributes (micro conecpts) such as name, dosage, dosage instruction, quantity, statement, form, request and manufacturer.

## 3. Annotation Guideline

The first criterion of developing an annotated corpus is to follow an annotation scheme and guideline to maintain consistency in annotation. The greatest challenge was to decide first what information should be extracted and propose a guideline to support the task. It is very important that all the annotators should annotate the documents in the same standard to minimize the errors and to avoid discrepancy. The first draft of the guideline was developed based on the initial analysis of randomly chosen 100 sentences. Annotation guideline had gone through multiple iterations of testing and revisions for updating new rules and concepts depending on the agreement and disagreement between the annotators to develop a quality corpus. After the first draft of annotation guideline, our annotators annotated another set of 100 sentences to examine the effectiveness of the guideline. The analysis of this annotation showed a considerable amount of agreement between the annotators. The updated version of guideline is ready now.

In the guideline we have tried to define all the concepts of the data model with examples and proposed some general annotation rules based on the initial analysis of the corpus. The first part of the guideline discusses the general rules for annotation. It is very important to decide first span of the text for annotation. We have tried to propose some rules for deciding the span of the texts. Here are few examples:

- Articles, adjectives, adverbs and all the modifiers and possessives should be excluded while choosing the text for annotation to decide more consistent span. We should not

include unnecessary modifiers such as ‘that’, ‘bad’, ‘special’, ‘your’, ‘my’. The span of the texts should be decided based on its relevance to the concepts.

- Multiword Expressions (MWE) is another challenging aspect such as “white discharge from vagina”, “frequent urge to urinate”, “trouble starting and stopping urination”. In these cases, the entire phrase should be annotated instead of a single string. Sometimes, we need to consider the whole sentence to understand the meaning and the aspect such as “My BP went up to to150/90 and 170/94.” The whole sentence should be annotated as observation-physiology to understand the problem patient is suffering from. If we just consider ‘150/90’ and 170/94’, the numbers can not specify it as blood pressure and more over as physiological observation. Therefore, the embedded concepts should not be annotated separately but together to express the right concern.
- Compound words also hold similar approach. Some compound words in our corpus are hyphenated and some are not. Therefore, it becomes difficult to decide the span of these kinds of texts. For example, “treatment plan”, “medical release”, “blood work”, “stress test”, “lab test” and “follow-up”, the meanings of these examples cannot be predicted from the individual expressions such as ‘medical’ , ‘stress’, ‘lab’ and ‘follow’ so we need to consider the whole phrase or the compound term.
- We have decided to remove all the private information related to patients, their family and practitioners such as name, contact details, identity number, clinic number, and social security number.
- Any information related to time and duration of appointment, surgery should not be annotated. They do not hold an integral part of information for our study.
- We have decided not to annotate any punctuation marks such as semi-colon, colon, hyphen, quotation marks and periods.

The second part defines all the concepts and rules for identification and extraction of those terms with some specific examples. We have already discussed that all the healthcare concepts in the data model have been based on the resources and elements of FHIR and all the 28 concepts have been organized and sub-categorized according to their scope and functions based on FHIR infrastructure for handling resources. There are few texts which are overlapping and it is difficult to categorize them under one concept. Our annotators have faced a lot of challenges due to this and they have disagreed in many contexts while annotating. Later, we have tried to resolve them through discussions and iterative process in annotation. Inter-annotator agreement does not only depend on the annotators but also on a well-defined guideline (For detailed information on annotation guideline see Supplementary document 3: Guideline for Annotating Patient Portal Secure Messages).

## 4. Annotation Process and Calculation of Inter-annotator Agreement

We chose Multi-purpose Annotation Environment tool [4] for annotating patient secured messages. MAE is an annotation tool for natural language annotation and it needs a task definition in the form of slightly customized Document Type Definition (DTD) files [4]. DTD files are used to define the tags and attributes for annotation and they can be easily created and modified. Once the DTD is created, the annotators can upload the DTD file and a file for annotation in the MAE tool. While we select and annotate a text the color of the text is changed

to the color associated with the tag. We will show it with some examples. We created a DTD file with 28 meso-concepts and 85 micro-concepts for annotation. Our DTD file had undergone few revisions after our guideline and data model were revised. While our annotators were annotating, they uploaded a single file or sentence each time and annotated it and saved it in xml format. If they need to revise the annotation later, they can open the xml file in the MAE tool and make the corrections. We chose MAE tool for its ease of use. We have cited few examples of annotation from MAE tool below.

Snippet: 1

*<?xml version="1.0" encoding="UTF-8" ?>*
*<-Annotation_v0.3>*
*<TEXT><![CDATA[Apparently my mother has a urinary tract infection...i wonder if it is the same issue as when she came to the clinic on jan 22-3?]]></TEXT>*
*<TAGS>*
*<ORGANIZATION id="O0" spans="100~106" text="clinic" />*
*<CONDITION id="C0" spans="20~43" text="urinary tract infection" attributes="name" />*
*</TAGS>*
*</Annotation_v0.3>*

Snippet: 2

<?xml version="1.0" encoding="UTF-8" ?>
<Annotation_v0.3>
<TEXT><![CDATA[He was very happy with my lipid panel so he is ready to drop my Lipitor to 10mg per day.]]></TEXT>
<TAGS>
<LAB-TEST id="D0" spans="28~39" text="lipid panel" attributes="name" />
<MEDICATION id="M0" spans="64~71" text="Lipitor" attributes="name" />$
<MEDICATION id="M2" spans="75~79" text="10mg" form="unknown" ingredient="unknown" attributes="dosage" />
</TAGS>
</Annotation_v0.3>

Snippet: 3

<?xml version="1.0" encoding="UTF-8" ?>
<Annotation_v0.3>
<TEXT><![CDATA[Since I am having surgery October 20, I need to reschedule my upcoming Remicade treatment at my local hospital.]]></TEXT>
<TAGS>
<ORGANIZATION id="O0" spans="98~106" text="hospital" />
<PROCEDURE id="PRO0" spans="18~25" text="surgery" />
<PROCEDURE id="PRO1" spans="67~85" text="Remicade treatment" attributes="name" />
</TAGS>
</Annotation_v0.3>

We have two annotators, a clinically trained linguist and a student pharmacist for the annotation. Both of them annotated same set of sentences independently. Individually single annotated document can reflect missing annotation, some idiosyncracies and consistent error. The level of agreement between the annotators is determined in terms of matches and non-matches between the annotators for each sentence. We used GATE software [5] for calculating Inter-annotator agreement (IAA). GATE software provides various tools for automatic evaluation of two annotated sets within a document. We need to change two sets of the annotated xml files to the format which should be supported by GATE software otherwise the information of annotators and the annotation sets will not be shown here. Once we upload the files there, we can choose the annotation type, annotation features and measures we need and can compare both the sets. The IAA scores were measured using the F1 score as a criterion. The F1 score measures the harmonic mean of precision and recall between the annotators using one annotator as a standard and the other as a reference [6]. The reson for applying F1 score compared to conventional measures such as Kappa is that F1 scores are computed at the level of entities [7]. This gives us a complete understanding of the span of the concepts on which annotators agree, disagree and partially agree. We followed the lenient parameter for measuring the IAA. With the lenient approach, the annotations which overlap, are counted as a partial match and on the contrary with strict approach, each annotation has to be a complete match with one another. The IAA scores were measured based on two annotation sets (each consists of 100 sentences). The agreement levels were determined for each concept. During the comparison between the two sets of the first set of annotation, the level of agreement between the annotators were consistent for the meso-concepts such as ‘appointment’, ‘diagnostic report’, ‘immunization’, ‘medication’, ‘practitioner’ and on the other hand, it was very unsatisfactory for the meso-concepts such as ‘specimen’, ‘related-person’, ‘document-reference’, ‘eligibility’, ‘healthcare-service’. We followed an iterative process of annotation, IAA calculation and discussion on agreements and disagreements to achieve a quality corpus.

### Challenges in Annotation Process

During the annotation process we faced a number of challenges and we discussed and designed some solutions how to handle those challenges.

- Noisy nature of language corpora makes the data detrimental for analysis such as typographical and spelling errors, irregular punctuation, informal and colloquial language use and abbreviations. It becomes difficult for researchers to extract valid and significant information out of it and map them to standard terminologies. In our study, we have tried to capture all the variations and irregularities of language use for generating a corpus for better understanding and machine learning.
- Idiomatic nature of language: Pre-constructed Multi-Word Expression (MWE) behaves like a single semantic unit. But sometimes it becomes difficult to select the span of those words. The meanings are not predictable from the properties of the individual lexemes in the MWE. They might be analyzed into single segments but they probably will not give us the right meaning of the concept. Therefore embedded concepts should not be annotated separately, such as “trouble

starting and stopping urination". The entire phrase should be annotated here instead of 'urination' to express the right concern.

- The nature of language in clinical domain is also difficult to process sometimes such as 'PTH', 'PC', 'MD', 'apt', 'inj'. Here 'PC' means primary care and 'PTH' means parathyroid hormone. We need to study the context very well to understand what it actually stands for. We tried to incorporate all the variations of language in order to train an efficient machine learning system.
- Semantic ambiguity is another aspect which needs careful attention. Semantic ambiguity occurs when a word has more than one interpretation. For example, "I was afraid the lab here wouldn't do the large kit because of the 'Dec only' phrase". In this example the word 'lab' might have two possible meanings: one is lab test and the second one is laboratory where the tests are done. We should need to consider the context here. The word, 'lab' here should be annotated as organization where the tests are done.
- Overuse of adjectives and adverbs often create a problem for automatic processing of a text and a system cannot always comprehend the boundary of the text right away with all the modifiers. So we have decided not to annotate adjectives, adverbs and all the modifiers.
- Our annotators found it very difficult to address the confusion between organization and healthcare service. We revised our guideline in order to make the concepts more clear and to avoid confusion. Organization is a formally organized and recognized body of people for providing healthcare service and healthcare service includes all the category or types of services available or provided by an organization and also the associated departments in the delivery of care to patients as allied health, emergency service, pharmacy, active rehab, social support, radiology department. Therefore 'NW Clinic Pharmacy' should be annotated as an organization but 'Holy Spirit School' should not because it is not a clinical organization. On the other hand, 'insomnia clinic' should be annotated as organization because it refers to a name of a clinic. If it refers to some specific building such as 'Baldwin 6 clinic' or 'Charlton' we should not annotate them.

## 5. Annotated Corpus

Figure S1 below shows the percentage of the macro and meso concepts in the annotated corpus.

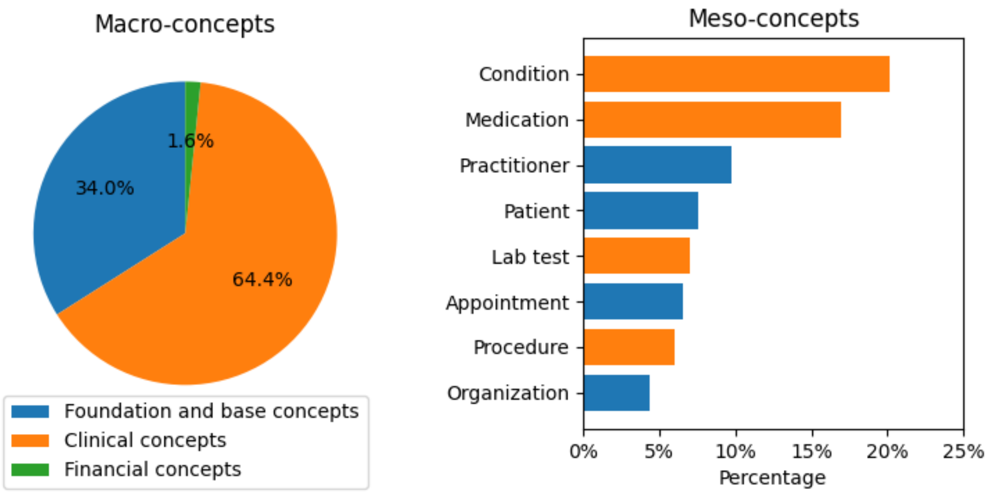


**Figure S1** Distribution of macro- and meso-concepts in the annotated corpus

## 6. Topic modeling

In this study, we chosen three frequently discussed health micro-concepts – fatigue, prednisone, and patient visit, as typical examples for topic analysis of relevant patient portal messages. We identified 41,490, 27,743, and 95,533 patient portal messages that mentioned the health micro-concepts (i.e., fatigue, prednisone, and patient visit) by using MetaMap [8] to examine the focus of those messages, respectively. We encoded multi-word concept with MetaMap, tokenized the sentences, removed stop words, and lemmatized each word. After that, we used a machine learning for language toolkit MALLET [10] and an impletmented state-of-the-art unsupervised topic-modeling method Latent Dirichlet Allocation (LDA) [9] to learn the hidden topics of patient secure messages related to each of three health micro-concepts (i.e., fatigue, prednisone, and patient visit). We determined the optimal topic number for three selected health micro-concepts by calculating the topic coherence at different topic numbers (i.e., 2, 4, 6, 8, 10, 12, 14, 16, 18, 20, 22, 24, 26, 28, and 30). As shown in Figure S1, we found that the optimal topic number was 8 for fatigue related messages, 12 for prednisone related messages, and 16 for patient visit related messages. We set the automatic optimization of the hyperparameters of $\alpha$ and $\beta$ in the MALLET during the topic modeling. $\alpha$ controls the topic distributions over a document and $\beta$ determines the word distributions over a topic.

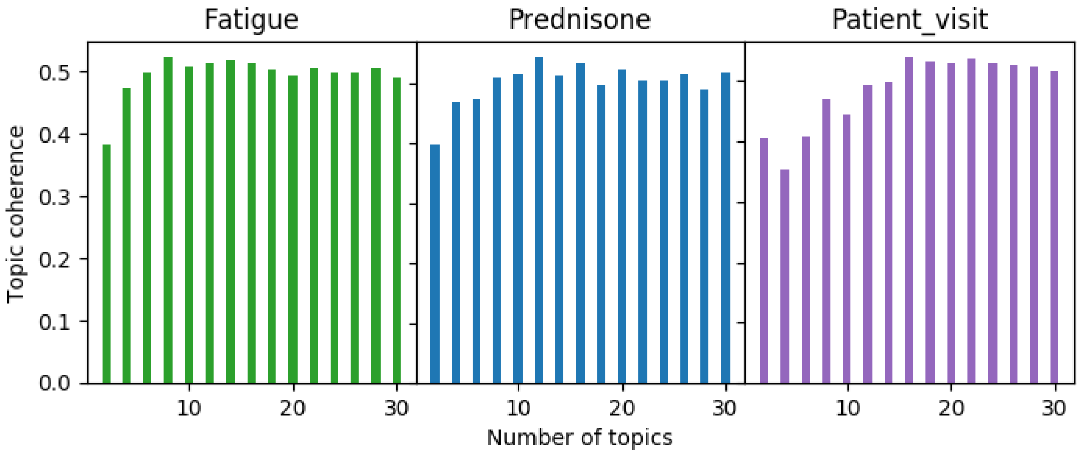


**Figure S2** Topic coherence in terms of topic count

We use a color scheme to represent the top micro health concepts in the topics of patient secure messages associated with fatigue, prednisone, and patient visit (See Figure 3 in the manuscript).

## Reference:


1. Health Level Seven International. *HL7 FHIR Release 4*. 2019; Available from: https://www.hl7.org/fhir/index.html.
2. Mayo Clinic. *Patient Online Services*. 2019; Available from: https://onlineservices.mayoclinic.org/content/staticpatient/showpage/patientonline.

3. Irizarry, T., A.D. Dabbs, and C.R. Curran, *Patient portals and patient engagement: a state of the science review.* Journal of medical Internet research, 2015. **17**(6): p. e148.
4. Stubbs, A. *MAE and MAI: lightweight annotation and adjudication tools*. in *Proceedings of the 5th Linguistic Annotation Workshop*. 2011.
5. Cunningham, H. *GATE: A framework and graphical development environment for robust NLP tools and applications*. in *Proc. 40th annual meeting of the association for computational linguistics (ACL 2002)*. 2002.
6. Gurulingappa, H., et al., *Development of a benchmark corpus to support the automatic extraction of drug-related adverse effects from medical case reports.* Journal of biomedical informatics, 2012. **45**(5): p. 885-892.
7. Blackman, N.J.M. and J.J. Koval, *Interval estimation for Cohen's kappa as a measure of agreement.* Statistics in medicine, 2000. **19**(5): p. 723-741.
8. Aronson, A.R. and F.-M. Lang, *An overview of MetaMap: historical perspective and recent advances.* Journal of the American Medical Informatics Association, 2010. **17**(3): p. 229-236.
9. Blei, D.M., A.Y. Ng, and M.I. Jordan, *Latent dirichlet allocation.* Journal of machine Learning research, 2003. **3**(Jan): p. 993-1022.
10. McCallum, A.K. *Mallet: A machine learning for language toolkit*. 2002; Available from: http://mallet.cs.umass.edu.
11. Aronson, A.R. and F.-M. Lang, *An overview of MetaMap: historical perspective and recent advances.* Journal of the American Medical Informatics Association : JAMIA, 2010. **17**(3): p. 229-236.
12. Röder, M., A. Both, and A. Hinneburg. *Exploring the space of topic coherence measures*. in *Proceedings of the eighth ACM international conference on Web search and data mining*. 2015. ACM.
