# Supplementary material for: Analyzing Patient Secure Messages Using a Fast Health Care Interoperability Resources (FIHR)–Based Data Model: Development and Topic Modeling Study
Source: J Med Internet Res. 2021 Jul 30;23(7):e26770. doi: 10.2196/26770 (PMC8367168; doi:10.2196/26770)
Supplement: Multimedia Appendix 3 [file jmir_v23i7e26770_app3.pdf]

**Analyzing Patient Secure Messages Using a Fast Health Care Interoperability Resources–Based Data Model: Development and Topic Modeling Study**

**Multimedia Appendix 3**

Amrita De[†1], Ming Huang[†1], Tinghao Feng[2], Xiaomeng Yue[3], and Lixia Yao[*1]

[1]Department of Artificial Intelligence and Informatics, Mayo Clinic, Rochester, MN, United States

[2]Department of Computer Science, University of North Carolina at Charlotte, Charlotte, NC, United States

[3]Division of Pharmacy Practice and Administrative Sciences, James L. Winkle College of Pharmacy, University of Cincinnati, Cincinnati, OH, United states

[†] Equal contribution

**Corresponding Author:**

**Lixia Yao, PhD**

All authors have no conflict of interest to declare.

## Introduction

In the US, government programs such as Medicare Access and CHIP Reauthorization Act (MACRA) [1] and the Medicare EHR Incentive Program, commonly known as "Meaningful Use" [2] have incentivized the growing adoption of Electronic Health Records (EHR) systems and Patient Heath Records (PHR) in a meaningful way to improve healthcare. EHR keeps track of patients' medical records and allows healthcare providers to diagnose patients in an efficient way and provides secure care to patients [3]. Along with EHR, many healthcare providers allows patients to access their healthcare records via online portal to engage patients in healthcare decisions and improve the healthcare quality. Online patient portal is a typical tethered system [3] and an extended version of EHR system which allows patients to access their medical records and communicate with their providers through secure messaging on wide range of health issues, diagnosis, appointments and prescription refills. But the analysis of the content of the large amount of secure patient messages has not been done systematically unraveling its potential for improving the healthcare quality, due to data privacy and lack of standard data exchange framework. In our study, we propose to develop a data model for patient secure messages based on Health Level Seven (HL7) Fast Healthcare Interoperability Resources (FHIR) [4] to understand, analyze the content of the messages and extract significant information from online patient portal. We aim to create an annotated corpus to further train and test a machine learning model to automatically parse patient secure messages.

We collected more than 2 million patient generated secure messages from Mayo Clinic Patient Online Services [5]. We randomly selected patient secure messages for creating the data model and annotated corpus. The first criteria of developing an annotated corpus is to follow an annotation scheme and guideline to maintain consistency in annotation. We created sets of 100 message sentences each for creating a well-defined annotation guideline. We developed the first version of our annotation guideline by analyzing of the HL7 FHIR and the sentences in the first set of message sentences. After that, our annotators annotated the second set of sentences to examine the effectiveness of the guidelines. We finalized the annotation guideline after an iterative process of discussions, agreements and disagreements between the annotators on annotation. Our data model has a 3-level hierarchical structure of health system concepts – macro concepts, meso- concepts and micro concepts. The annotation guideline defines all the concepts very clearly and simple way to lessen the ambiguity and error in annotation and develop a quality corpus. Our guideline has two parts – the first part discusses the general instructions for annotation and the second part provides us with the definition and rules for identifying and extracting the healthcare concepts. The annotation guideline is discussed in details below:

## Part I

## General Instructions for Annotation

The purpose of our study is to analyze and extract those healthcare relevant concepts in patient secure messages which the patients communicate with their healthcare provider frequently, in

order to better understand their concern and evaluate the scope of improvement in the overall healthcare system. We used HL7 FHIR standard based data model to develop the guideline in order to create annotated patient secure messages. The guideline outlines all the rules specific to meet the goal of our study. The first part specifies some over all general rules to follow.

1. We should decide first the span of the texts for annotation. We determine some rules for deciding the span of the texts for annotation as follows:

    - No articles should be annotated as a concept or part of a concept, e.g., “My doctor is sending me for a colonoscopy this week”, “We left our appointment before I got the Vanderbuilt assessment for my daughter’s music teacher”; in the mentioned examples we would take ‘colonoscopy’ and ‘Vanderbuilt assessment’ as a text for annotation but not the preceding articles ‘a’ and ‘the’. Our aim is to find out the relevant texts associated with specific concept, concerning patients.
    - Adjectives, adverbs and all the modifiers and possessives should be removed while selecting texts for annotation. We should not include unnecessary modifiers and possessives, e.g., “this medicine”, “that surgery”, “this pain”, “bad stomach pain”, “my rheumatoid arthritis”, “special calcium pill”, “new prescription”. Here all the modifiers such as ‘that’, ‘this’, ‘bad’, ‘my’, ‘special’, ‘new’ should not be considered as texts to be annotated.
    - Multiword Expressions (MWE) can be another challenging area which deserves to be handled properly. The typical examples are “white discharge from vagina”, “frequent urge to urinate”, “trouble starting and stopping urination”, “down lower in my digestive symptoms”. In such cases the entire phrase should be annotated. The single string such as ‘discharge’, ‘vagina’, ‘urinate’, ‘urination’, ‘symptoms’ would not be able to do justice to express the right concern as well as the concept. Sometimes, we should have to consider the whole phrase or sentence to understand the meaning and context. For examples, “ His thyroid is going to in and out of hypo-hyper” and “My BP went up to to150/90 and 170/94” – the whole sentence should be annotated as “condition-symptom” and “observation-physiology” to understand what the patient is trying to communicate or share. If we just consider ‘150/90’ and ‘170/94’- the numbers cannot determine it as blood pressure and more over as a physiological observation.
    - Another important concern is how to deal with the compound words. There are some compound words in our corpus. Sometimes, it becomes difficult to decide the span these texts. For instance, “treatment plan”, “stress test”, “blood work”, “lab test”, “drooping hemoglobin”, “kidney function”, “radiology report”, “medical release”, and “dental work” – apparently all these words seem to have separate meanings but contextually they refer together a single concept. They s should be annotated together. The texts ‘medical’ and ‘release’ would separately take different tags and separate meaning but ‘medical release’ is a compound term referring some healthcare document. Like MWE, the meanings are not predictable from the individual expressions so we need to consider the whole phrase or the compound term. There are some other examples such as “follow up”, “follow-up visit” which should be annotated as a single concept as well.

2. The language of patient narrative is very ambiguous and different from the clinical language. Patients often use colloquial and informal language and expression to share their condition and experience. It becomes a challenge for the researchers to understand and extract significant information out of it and map them to the standard clinical terminologies. For example, “For last few days and into today my throat has been very dry, scratchy, swollen, hard to swallow and very painful… it really hurts to swallow, I cannot swallow.” In this example the patient shares his condition in a very informal way by using nonmedical terms such as “dry”, “scratchy”, ‘hard to swallow”. In few cases, patients just use “urine”, “cholesterol”, “sugar” instead of using the name of the specific lab test. We need to analyze and extract all these information very carefully depending on the context.

   Semantic ambiguity is another aspect which needs careful attention. Semantic ambiguity occurs when a word has more than one interpretation. For example: “I was worried that the lab here wouldn't do the large kit because of the “Dec only” phrase”. In this example the word “lab” might have two possible interpretations - one is lab tests and the other is laboratory where the tests are done. We should need to consider the context to understand which meaning is intended. The word “lab” here should be annotated as organization where the tests are done.

   The spelling error, typographical error and use of abbreviation can also make the text detrimental for analysis and lowers the quality of data and it becomes less accessible to automated processing by a computer. We need to deal with them very carefully. There are few examples such as:

   - Spelling error – “I have been working with the trainer on specific exeercies, I am working with her 3 times a week under her guidaance and am in the pool 2-3 times a week doing exercisies recommended by her.”
   - Typographical error – “ifnotdue for papsmear need to renew med”
   - Abbreviations – “BP” for Blood Pressue, “RA” for Rheumatoid arthritis, “ob/gyn” for obstetrician/gynecologist.

We aim to capture all the language deviations in our corpus so that any kind of patient secure messages can be parsed without an error automatically in future.

3. We decide to keep all the private information related to patient such as patient’s name, contact information, identity number, clinic number, Social Security Number (SSN), information related to gender, marital status as patient privacy and not to be disclosed.
4. Similarly, all the information related to practitioner such as name, contact information and other private information should be kept secured and not disclosed.
5. The address of any clinic or hospital should be annotated together as a single entity instead of annotating them separately as street name, city or pin, such as “5 Main Street, Albany, NY” should be annotated together as organization’s address.
6. All the generic terms such as “doctor” or “Dr.”, “specialist”, “patient”, “treatment”, “report”, “test”, “surgery”, “labs”, “blood work”, “blood test” and “pharmacy”,

"medicine" should be annotated. They are very significant to understand those aspects patients are concerned about.

7. We agree not to annotate any information related to date, time and duration for the appointment or e-visit or symptoms of any diseases. This information doesn't concern our study.
8. Punctuation marks should not be annotated such as semi-colon, colon, hyphen, quotation marks, and periods.

**Part II**

The second part of the guideline defines all the concepts of the data model and further categorizes and explains them with examples for identifying and extracting in a better way. The concepts in the data model have been grounded on the resources and elements of HL7 FHIR. Data model has 3 macro-concepts (foundation and base concepts, clinical concepts, and financial concepts), 28 meso-concepts and 85 micro-concepts. We decide to add a micro-concept "unspecified" as an attribute under most of the meso-concepts to deal with the general terms. "Unspecified" concepts are those general meso-concepts which cannot be further assigned to any specific micro-concepts. They have been discussed in details with definition and examples for how to identify, analyze and extract them. We consult and follow the definition and description provided by FHIR to define the concepts [4].

**Foundation and Base concepts**: Foundation and base concepts are the basic infrastructure of a healthcare system on which rest of the specifications is built. There are 9 meso-concepts under foundation and base concepts. They have discussed below along with their respective micro-concepts.

1. **Patient**: Patient is an individual or animal receiving healthcare. Patient is further categorized as 4 micro-concepts:
   - Patient-unspecified: Unspecified refers to the general texts such as "patient" which do not qualify for any micro-concepts under the meso-concept "patient". These terms should be annotated as unspecified.
   - Patient – privacy: All the personal, demographic and administrative information fall into this category and we decide to keep it as private information and they should not be disclosed for data protection and privacy issue. For example, any personal information such as name, gender, birthdate, marital status; any confidential information such as clinic number, social security number (SSN), driver's license number should be annotated as patient privacy. Only the text "patient" should be annotated under unspecified, such as "I am a 'patient' of … the surgery in the hospital" and rest of the patient related information should be kept under privacy. We will not disclose any secured information related to patients in our result and paper.
   - Patient – lifestyle: Patient's lifestyle and personal way of living should be annotated as patient-lifestyle, e.g. "I never started taking the prescribed medicine….. if I could manage my cholesterol and triglycerides again through diet and exercise". "Diet and exercise" in the sentence should be annotated as patient-lifestyle.

- Patient – diet: Patient's food habit and diet should be annotated separately as they can add important information to patient record, e.g. "The last time we met with the doctor, he suggested cranberry concentrate and vitamin c to keep her urine/acidic to keep bacteria from growing" – "cranberry concentrate" should be annotated as diet here.

2. **Practitioner**: Practitioners are all those individuals who are engaged in treatment, healthcare processes and services and hold formal responsibilities. Practitioners include physicians, surgeons, nurses, radiographers, pharmacist, physician's assistant, laboratory technicians and assistants, receptionist at the information and registration desks, precisely all the healthcare workers. Every practitioner has certain defined role in the healthcare system. The practitioners' attributes are discussed below as micro-concepts:
   - Practitioner – unspecified: We decide not to annotate practitioner's name. Only the text "doctor", "Dr.", "nurse", "specialist" and "provider", should be annotated under unspecified as they cannot be categorized under other attributes e.g. "Is he just mad at me cause I have elected to find a different 'doctor' closer to me?", "Hi *****, I forgot to send you a message after my appointment with 'Dr.' ***** last week", "For insurance reasons, …, so we are flexible if specialist schedules are tight.". In the above sentences the texts "doctor" and "Dr.", "specialist" should be annotated as practitioner.
   - Practitioner – privacy: Any contact details for practitioners such as telephone numbers, email id, personal address should be annotated as private information. As they are secured information and we should not disclose it in our result.
   - Practitioner – specialty: Practitioner's specialty is an important aspect to understand the specific area the practitioner is associated with such as "cardiologist" , "radiologist", "gynecologist", 'pulmonologist" and so on. In the following sentence, "I was wondering if you would be able to refer me to a good marriage therapist ?" - the text "marriage therapist" is to be annotated as a practitioner's specialty.

3. **Related Person**: Related person is a person involved in the care for a patient but not the target of healthcare. They are non-healthcare-specific professionals and do not hold any formal responsibilities. They serve as a contact person for the patients and often a source of information about the patients. Examples of related persons are patient's wife or husband, patient's relatives or friends, patient's parents or children, a neighbor bringing a patient to the hospital, a patient's attorney or guardian etc.
   - Related person – unspecified: We create unspecified as a micro-concept under related person to identify the terms which refer to related person but do not necessarily fall under any other micro-concepts.
   - Related person – relationship: Related person relationship indicates the kind of relationship between the related person and the patient. For an example, "In my family, my mother had diabetes, and father had thyroid Dz" in this sentence "mother" and "father" is related person whose medical history is also important in the treatment of the patient.
   - Related person – telecom: It refers to the contact details of the related person.

4. **Organization**: Organization is a formally organized and recognized body of people for providing healthcare service. We  propose some micro-concepts for organization as follows:
   - Organization- unspecified: All the general terms referring organization such as “hospital”, ‘pharmacy”, “lab” and “clinic” should be annotated under as unspecified.
   - Organization – name: The name of the organization helps us to identify the organization across multiple systems, e.g. “NW Clinic Pharmacy” should be annotated as an organization but “Holy Spirit school” should not because it’s not a clinical organization. On the other hand, “insomnia clinic” should be annotated as organization because it is referring to a name of a clinic. If it refers to some specific building name of an organization, we should not annotate it because it is very much contextual such as “Baldwin 6 clinic” or “Charlton (1-13)”. We do not need such detailed information for our study.
   - Organization – contact number: All the contact details such as phone number, fax number of an organization should be annotated as part of the significant information.
   - Organization – address: If the address of the organization is mentioned in the narrative, it should be annotated.

5. **Healthcare Service**: The healthcare service includes all the category or kinds of services available or provided by an organization and also the associated departments in the delivery of care to a patient. There are few examples of healthcare services: allied health, emergency services, pharmacy, drug and alcohol service, 24 hours crisis telephone counselling support, active rehab, and social support, radiology department, neurology department. The healthcare services have been classified further so that relevant information can be extracted.
   - Healthcare Service – unspecified: All the general terms related to the healthcare service should be annotated under unspecified.
   - Healthcare Service – name: Healthcare service name includes the services and associated departments of an organization at a location. For an example: “I would like my prescription sent to the mayo mail order pharmacy as I have mentioned in my profile” and “ … your radiology department should know how to get access to it or open it,….” – in these sentences “mayo mail order pharmacy’ and “radiology department” should be annotated as healthcare service name.

6. **Appointment:** Appointment refers to the booking of a healthcare event among patients, practitioners, related person and devices for a specific time The  significant attributes in appointment are discussed below:
   - Appointment – unspecified:  All the texts such as “appointment”, “appt”, “apt”, “visit”, and “consultation” should be annotated under appointment unspecified. They do not specifically fall under status, type or reason and just provide general information.

- Appointment – status: Appointment status reveals the information whether the appointment is proposed, pending, booked, cancelled or no show.
- Appointment – type: Appointment type refers to the type of appointment service booked. Any kind of visit or appointment such as "follow up visit", 'e-visit", "child checkup", "electronic consult", "surgical GYN consultation", "treatment follow-up appointment" should be annotated. But there are exceptions such as "I have recently requested for an appointment with my primary care, but haven't heard yet". In this sentence "follow up" is an appointment type but it refers to just a response from other side.
- Appointment – reason: Appointment reason is why the appointment has happened or why the appointment is going to be performed.

7. **Device:** Device is the medical and non-medical device used for healthcare.
   - Device –name: Device name gives information on the device such as "artificial ligament", "biliary tube" and "tampon". They should be tagged as device-name.

8. **Encounter:** An encounter is an interaction between patient and healthcare provider for providing healthcare. In our data model the encounter has only one attribute as "type". An encounter-type can refer to pre-admission, admission, stay and discharge, e.g., "Hey **** I will be discharged tomorrow." In this sentence, 'discharged' should be annotated as encounter. There is an exception of this rule in the following sentences - "I am currently experiencing discharge that is not very comfortable" or "….. profuse yellow discharge that glues her eyes ……..". Here the text 'discharge' in both the sentences, is not a type of encounter but it refers to the symptom of a clinical condition. Therefore, before annotating any concept we need to understand the context very well. Same word might have multiple usages depending on the contexts. We try to capture different usage of colloquial language in our corpus.

9. **Document Reference:** Document reference includes formal patient centric documents and records, clinical notes, scanned paper and non- patient specific documents like policy paper. It has only one micro-concepts as- type. There are few examples for better understanding of the document reference such as "I need a copy of my father's medical records from August 2010-present", "I was wondering if I could get a healthcare summary and an immunization record from my healthcare provider", "I was told to submit a request and hold on to my sister's medics write up/synopsis", "Please let me know where and to who I can submit the medical history of my mother". In the above sentences, 'medical records', 'immunization record', 'medics write up/synopsis', 'medical history' are the examples of document reference which should be annotated. We find some more examples of document reference through our analysis of the narrative such as "Psychiatry and Addiction notes", "clinical notes and correspondence", "hx" , "form for release" etc. Here "hx" is the abbreviated term, referring to medical history.

**Clinical Concepts:** Clinical concepts are those concepts referring to core clinical problems, diagnosis procedure, medications, and care plan and so on for a patient. The meso-concepts under clinical concepts are discussed below.

10. **Allergy Intolerance:** Allergy intolerance is the risk of harmful or undesirable, physiological response which is unique to an individual as an exposure to a substance. We decide not to create an unspecified category for allergy intolerance. It is further classified as:

- Allergy Intolerance – name: Allergy Intolerance name refers to the name of the allergy the patient encounters.
- Allergy Intolerance – category: Allergy intolerance category refers to the types or categories of the allergy such as the allergy is from food or medication or from environment or biologic.
- Allergy Intolerance – criticality: Allergy intolerance criticality defines how critical the allergy is such as low, high or unable to access.
- Allergy Intolerance – asserter: It defines the source of the allergy.
- Allergy Intolerance – last occurrence: Last occurrence gives information on last known occurrence of a reaction.

11. **Adverse Event**: Adverse events occur during the course of medical care or medical research that have the potential of results in death. Adverse event can be classified further as:
- Adverse Event – unspecified: We create this micro-concept to label those text related to adverse event or the text "adverse event" itself which we cannot name otherwise.
- Adverse Event – name: An adverse event is any untoward medical occurrence in a patient or clinical investigation subject administered a pharmaceutical product (medication, devices so on) e.g., adverse drug event include preventable medication errors such as an accidental overdose or providing a drug to the wrong patient or non-preventable adverse drug reactions, such as an allergic reaction. In the following example "The doctor has taken me off the oral iron supplements given the constipation and the aforementioned bowel obstruction, I am experiencing for last few days" – "constipation" and "bowel obstruction" both are example of adverse event caused by the iron supplements.

12. **Condition**: Condition is the clinical problem or other event or situation which deserves to be treated. We can further categorize it for our convenience to extract relevant information about it.
- Condition – unspecified: This covers all the general texts related to condition but cannot be classified as name, context, onset, and symptom.
- Condition – name: Condition name identifies the name of the clinical condition, disease or problem e.g., "My family doctor certainly knows my past history with rheumatoid arthritis ….", "Last month I had bad stomach pains which… ", "…… my doctor suspects that the limb in my breast is cancer". In the above examples

“Rheumatoid arthritis”, “stomach pains”, “cancer” are referring to conditions and should be annotated as condition name.

- Condition – context: Condition context can be the reason for which clinical problem starts; e.g., “During my last visit (in June 2012), I had indicated I wasn’t interested in following up with a surgery consult related to the Morel-Lavaillee lesion from my fall last September.”- in this sentence “from my fall” should be annotated as context.
- Condition – onset: Condition onset identifies the estimated or actual date or time of its occurrence. For example: “urine stream dark throughout”, “strong urge to urinate present for 1 to 7 days”- “throughout” and “1 to 7 days” should be marked here as condition onset.
- Condition – symptom: Symptom is a subjective evidence of any disease, experienced by any individual, such as “ urine stream dark throughout” , “ frequent urge to urinate”, “sore bottom” , “limb in my breast”, “bp is up”, “right arm is numb” are examples of symptoms which should be annotated under condition-symptom.

13. **Procedure**: Procedure means an action performed on a patient such as physical intervention like an operation or less invasive like counselling or hypnotherapy. This refers to any kind of therapeutic, surgical or implant procedure, diagnostic procedures, physiotherapy, personal support services.

- Procedure – unspecified: General terms related to procedure such as “procedure”, “surgery”, “therapy” should be annotated under unspecified.
- Procedure – name: Procedure name identifies the procedures performed on patients such as “If papsmear is not due, I would like to renew med”, “The doctor cleared me for removal of biliary drain, since the ostomy had been sealed for 2 weeks”, “checking to see if I am able to get a complete physical from my primary care physician… I will have surgery there in next week”, “The doctor also was happy with the stress results”, “I took the occipital nerve injection yesterday and I am feeling no relief from it”. In the above sentences, “papsmear”, “removal of biliary drain”, “ostomy”, “complete physical”, “stress test” and “occipital nerve injections” refer to the names of the procedures and the text “surgery” should be annotated as procedure-unspecified.
- Procedure – report: Procedure report can be any report resulting from the procedure such as “colonoscopy results”.
- Procedure –follow-up: Follow-up refers to the follow-up visits after any procedure or surgery to monitor the improvement or deterioration in patient’s health.

14. **Family Member History**: Family member history records significant health conditions for a particular individual related to the patient in the context of care.  It is further categorized into micro-concepts such as :

- Family Member History – name: Name refers to the name of the family member.
- Family Member History – relationship: Relationship refers to the relationship with the subject for treatment, e.g., “In my family, my mother had diabetes, and father had thyroid DZ”.

- Family Member History – gender: Family member history- gender identifies the related person whose history is under concern, is male, female, other or it's unknown.
- Family Member History – age: It gives the information about the approximate age of the person.
- Family Member History – deceased: It reveals whether the family member is still alive or dead.

15. **Observation:** Observation is measurements and simple assertions made about a patient, device or other subjects. Observations can be vital signs such as body weight, blood pressure; laboratory data, clinical findings, personal and physiological characteristics, social history etc.
    - Observation – unspecified: There are few texts which are related to observation but not specifically refer to any other micro-concepts under observation.
    - Observation – physiology: Observation physiology includes the details and measurements of physiological aspect of a patient such as blood pressure, body weight, temperature, respiration rate, eye color and sensory observations. There are few examples: "We haven't noticed any problems with my son's speech or hearing per se , ….", "I don't have any difficulty in swallowing softer foods", " I want to ask about night time urination and it is causing me trouble". In all the cited examples, "speech or hearing", "swallowing softer foods" and "urination" are physiological observations and we should annotate them accordingly. Some more examples include: "I can also feel pulse in my ear", "I regularly get dizzy…. but I think this is due to my relatively low blood pressure". In both the sentences "pulse" and "low blood pressure" are examples of physiological observations.
    - Observation – value: Observation value represents the actual result of any physiological observation such as "pulse rate 68", "blood pressure 120/80", "blood pressure is so low", "BP went up to 140/80 and 162/91" etc. The whole phrase should be annotated as observation value to understand the context.

16. **Lab-Test**: Lab tests are performed on patients, groups of patients; and specimens are derived from them for particular investigation. Lab tests generally include clinical chemistry, hematology, microbiology, pathology, histopathology and related discipline. The micro-concepts for lab tests are discussed below:
    - Lab-Test – unspecified: We decide to label all the general terms related to lab-test under unspecified.
    - Lab Test – name: Lab test name identifies the tests performed on the patient such as "….. if I could manage my cholesterol again through diet and exercise and without any medication", "He says it's not unusual …… the prescribed medicine would help to get the calcium up", "Do you want me to do a 24 hr urine and FLC etc in next week?" – in the above examples, "cholesterol", "calcium", "urine and FLC" are examples of lab test.
    - Lab Test – result: In the following sentences, "Hi Dr. **** I have some questions regarding my cholesterol results from last week" and "… The lab normal range is 0 thru rsv negative" – "cholesterol result" and "rsv negative" are an example of lab test result and all the lab results should be annotated with this tag.

17. **Specimen**: Specimen is taken from biological entity living or dead for diagnostic analysis. Specimen includes cellular molecules, cells, blood and its components, tissues, body fluids, embryos and body excretory products. Specimen has only one micro-concepts as "name". Few examples are cited here: "Her stools have been pudding consistency for last few days and……harder", "I have had to PAP's after the surgery and it showed low grade squamous intraepithelial cells in the report", "…… the processing of biopsy specimens …". "Stools", "squamous intraepithelial cells", "biopsy specimens" – should be annotated as specimen-name.

18. **Body Structure**: Body structure is the anatomical structure of the subject receiving care. It contains details about the anatomical location of a specimen or body part which is under observation or undergoes any procedure or treatment. In the following examples, " …… if I wanted to keep my ovaries or not.", " There is rash on both armpits……..", " photos of retina", "…… when I swallow much of the time on the right side of my neck level with my Adams apple" – "ovaries", "armpits", "retina", "neck", "Adams apple" refer to body structure. They should be annotated under body-structure – name.

19. **Imaging**: Imaging study refers to X-Ray, computed tomography (CT) scan, ultrasound and magnetic resonance imaging (MRI) which provides information on particular clinical condition based on images produced in single study. Imaging study can be further categorized as:
   - Imaging – unspecified: All the general terms related to imaging should be annotated under unspecified.
   - Imaging – name: It identifies the study performed on the subject e.g. "There is no reference from Dr. **** about radiology report after x-rays were taken after my surgery last week", "…. Just got a call from breast imaging", "Dr. **** has set up an appointment for me for a mammogram next week", "EMG Autonomic testing". Here "x-ray", "breast imaging", "mammogram" and "EMG" should be annotated as imaging name.

20. **Medication:** Medication includes the identification and definition of a medication for the purposes of prescribing, dispensing and administering a medication as well as for making statements about it.
   - Medication – unspecified: All the general terms such as "medication", "medicine", "meds" should be annotated under medication - unspecified.
   - Medication – name: Medication name identifies the name of the medications which have been prescribed to the patients such as "Copaxone", "Prednisone", "Lialda" and "Imuran" etc.
   - Medication – dosage instruction: It gives instruction on how the medication should be taken.
   - Medication – quantity: Medication quantity refers to a specified amount of medication prescribed for the patient. For an example: "The doctor has prescribed me a total of 4 capsules 20 mg' and "I have taken 5 pills but my cough is still very

irritating and present". In these sentences, "4 capsules" and "5 pills" should be marked as medication quantity.

- Medication – dosage: Medication dosage refers to the details of how medication is taken or should be taken, e.g. "18 or 20 mg of prednisone"- here the dosage "18 or 20 mg" should be annotated as medication dosage.
- Medication – statement: Medication statement is a record of medication that indicates that the patient may be taking the medication now or has taken the medication in the past or will be taking the medication in the future e.g., "The doctor has prescribed me the new prescription after surgery but I am trying to manage my cholesterol again through diet and excercise", "I think I have mentioned to the nurse during a visit that I am not taking that medication and asked her to remove it from my list of medications". The texts "prescription" and "list of medications" should be annotated as medication statement.
- Medication – form: Medication form is the details about the medication form whether it's in oral form or shots or injections. For an example, "Doctor, I just wanted you to know that the injection in my finger helped to reduce the pain". In this sentence the text "injection" should be annotated as medication form.
- Medication – request: Medication request covers all type of orders for medications for a patient. This includes inpatient medication, community orders, over the counter medications. It may be used to support the order of medication-related devices. In the following sentences "My primary care was the last physician to put me through a refill request on this last Decemeber" and "Follow-up to Imitrex refill return message" where "refill request" and "Imitrex refill return message" should be annotated as medication request.
- Medication –manufacturer: It gives information on pharmaceutical companies who manufactures medicine.

21. **Immunization:** Immunization describes the event of a patient being administered a vaccine or a record of an immunization as reported by a patient or clinician or another party.
    - Immunization – unspecified: If there is any general term like "vaccine" that should be annotated under unspecified.
    - Immunization – name**:** Immunization name identifies the vaccinations which have been prescribed to the patients, such as chickenpox(varicella) vaccine, Hepatitis A vaccine (HepA), Hepatitis B vaccine (HepB) as well as Diphtheria, tetanus, Pertussis vaccine (DTaP), flu shots, shingles vaccine and Humira shot.
    - Immunization –date: Immunization date is the time when the immunization is performed.
    - Immunization –manufacturer: Immunization manufacturer gives information on vaccine manufacturer.
    - Immunization –site: Site is the bodysite where the vaccine is administered.

22. **Care Plan:** Care plan describes the intention and plan of course to deliver a care for specific condition to a patient by a practitioner for a period of time. For an example, "Monday I will go back to the surgeon to find out what type of cancer I have and what treatment recommendations I should follow.", "We are working with

psychiatrist on some behavior modification strategies since last 2 months". In both sentence "treatment recommendations" and "behavior modification strategies" should be annotated as care plan name.

23. **Referral:** Referral is when a doctor refers to the patient under treatment to a specialist or to other care team for better evaluation or to continue with the further treatment procedure. Here in the sentence "If my previous doctor won't send a referral letter directly to my new doctor, maybe he can send my medical reports to me". The text "referral letter" should be annotated under referral unspecified as it doesn't further refer any specific details.

24. **Risk:** Risk captures the predicted outcome for a patient, such as risk of health outcome as heart attack, particular type of cancer on the basis of lifestyle factors or family history or list of potential health risks based on patient's genetic analysis.

**Financial concepts:** The financial concepts cover financial transactions, billing, eligibility, and claims, payments which happen between a healthcare provider, patient and insurers. There are four meso-concepts, discussed in details below.

25. **Coverage-Eligibility:** Coverage-Eligibility category provides information on patient, insurer, insurance coverage, plan details, reimbursement and payment for healthcare service including both insurance and self-payment etc. It is further categorized into some micro-concepts:
    - Coverage-Eligibility – unspecified: It covers all the general terms which do not fall under other micro-concepts such as percentage, insurer and insurance. For example, "I would like to know what would my covered cost for a shingles vaccine and will there be a visit cost in addition to the vaccine". Here 'covered cost' and 'visit cost' should be annotated as coverage-eligibility-unspecified.
    - Coverage-Eligibility – percentage: In the sentence "The date of service has no impact on the benefit percentage; however, it is processed and sent by the lab until next week". The "benefit percentage" should be annotated as percentage.
    - Coverage-Eligibility – insurance id: Insurance id identifies the primary insurance holder for the coverage, e.g., "They have a copy of my insurance card and I just hope they would verify the correct insurance information before submitting the claim". Here "insurance card" should be annotated as insurance id.
    - Coverage-Eligibility – benefit-category: Eligibility benefit category gives details about all the benefits.
    - Coverage-Eligibility - insurer: Eligibility insurer refers to the insurer who issues the coverage such as "Cigna" "Medicare" etc. refer to the insurer and should be tagged as insurer.
    - Coverage-Eligibility - insurance: Coverage-Eligibility insurance gives the details about the insurance for example "mail order Rx" should be annotated as insurance.

26. **Claim-Payment:** Claim-Payment category is a list of healthcare services and products which have been provided or are to be provided to a patient and sent to the insurer for reimbursement and it also gives information on the status of payment for healthcare services e.g.
    - Claim-Payment – unspecified: All the general texts should be labeled under unspecified, e.g., "The Veteran affairs will verify and submit a claim on my behalf" – here "claim" should be annotated under unspecified.
    - Claim-Payment – total: Claim total is the total claim cost of the coverage.
    - Claim-Payment – billing-code: Billing-code is the code that identifies the charges e.g., "Please proceed with all the testing if the billing code can be medically necessary for filing claim" – here "billing code" should be annotated as claim billing-code.
    - Claim- Payment – balance: Payment balance is about how much is in the account e.g., "The balance on the account is $370 please contact our office to discuss payment procedure". In this sentence 'balance on the account' should be annotated under balance category.
    - Claim- Payment – description: Payment description describes explanation of purpose and use. In the following sentence, "After reviewing the Explanations of Benefits (EOBs) from Blue Cross Blue Shield, I see that balances of $656 and $266 for the processing of the biopsy specimens (specimen) on 3/16/14 is not a co-pay assessed by your plan because you were not here on that day. It is the normal co-insurance amount because you do not have a 100% plan benefit until your yearly out of pocket maximum is achieved". The "co-pay' and "co-insurance" should be annotated as claim-payment description.

27. **Account:** Account is financial tool for recording charges, payments and adjustments and holds information about who are responsible for payments. It is further categorized into two micro-concepts – unspecified, identifier-privacy. If we find any private information such as patient's account details, we should keep them under account attributes 'identifier privacy' and general information should be tagged under unspecified.
28. **Explanation of Benefits:** Explanation of Benefits gives information on claim details, adjudication details from the processing of a claim and sometimes, it holds information on the accounts, balances for informing patients of the benefits to be provided. In the following sentences, "After reviewing the Explanations of Benefits (EOBs) from Blue Cross Blue Shield, I see that balances of $656 and $266 for the processing of the biopsy specimens (specimen) on 3/16/14 is not a co-pay assessed by your plan because you were not here on that day. It is the normal co-insurance amount because you do not have a 100% plan benefit until your yearly out of pocket maximum is achieved" and "I will send you copies of the EOBs for your future record". 'Explanations of Benefits' and 'EOBs' should be annotated under explanation of benefits-unspecified because they don't provide any detailed information.

In the data model there are some micro-concepts for which do not find any example texts for annotation but we decide to keep them in the data model in order to annotate the large amount of

data with wide range of information in future. This annotation guideline can be used to develop annotated patient secure messages in large scale for future study. We believe that the data model and annotated corpus would help us to understand and extract the concepts that are frequently concerning to patients and their opinion towards the quality of healthcare received, allowing us the opportunity to improve individualized patient care.

## Reference


1. Casalino, L.P., *The Medicare Access and CHIP Reauthorization Act and the corporate transformation of American medicine.* Health Affairs, 2017. **36**(5): p. 865-869.
2. Blumenthal, D. and M. Tavenner, *The "meaningful use" regulation for electronic health records.* New England Journal of Medicine, 2010. **363**(6): p. 501-504.
3. North, F., et al., *Impact of patient portal secure messages and electronic visits on adult primary care office visits.* Telemedicine and e-Health, 2014. **20**(3): p. 192-198.
4. Health Level Seven International. *HL7 FHIR Release 4*. 2019; Available from: https://www.hl7.org/fhir/index.html.
5. Mayo Clinic. *Patient Online Services*. 2019; Available from: https://onlineservices.mayoclinic.org/content/staticpatient/showpage/patientonline.
